# Coagulation dysfunction in ICU patients with coronavirus disease 2019 in Wuhan, China: a retrospective observational study of 75 fatal cases

**DOI:** 10.18632/aging.202223

**Published:** 2020-12-09

**Authors:** Jiaran Shi, Wang Zhang, Ling Sang, Zhaohui Qu, Ming Zhong, Li jiang, Bin Song, Liang Kang, Yun Zhang, Xingxiang Wang, Dingyu Zhang, Xia Zheng

**Affiliations:** 1 Department of Cardiology, The First Affiliated Hospital, Zhejiang University School of Medicine, Hangzhou, Zhejiang, PR China; 2 Department of Infectious Diseases, Sir Run Run Shaw Hospital, Zhejiang University School of Medicine, Hangzhou, Zhejiang, PR China; 3 Department of Critical Care Medicine, The First Affiliated Hospital of GuangZhou Medical University, GuangZhou Institute of Respiratory Health, Guangzhou, Guangdong, PR China; 4 Department of Critical Care Medicine, Jinyintan Hospital, Wuhan, Hubei, PR China; 5 Department of Critical Care Medicine, Zhongshan Hospital, Fudan University, Shanghai, PR China; 6 Department of Critical Care Medicine, Xuanwu Hospital, Capital Medical University, Beijing, PR China; 7 Department of Tuberculosis and Respiratory Disease, Jinyintan Hospital, Wuhan, Hubei, PR China; 8 Department of Critical Care Medicine, The First Affiliated Hospital, Zhejiang University School of Medicine, Hangzhou, Zhejiang, PR China; 9 Research Center for Translational Medicine, Wuhan Jinyintan Hospital, Hubei, PR China; 10 Joint Laboratory of Infectious Diseases and Health, Wuhan Institute of Virology and Wuhan Jinyintan Hospital, Chinese Academy of Sciences, Hubei, PR China

**Keywords:** coronavirus disease 2019, COVID-19, death, coagulation dysfunction, inflammation, cardiac injury

## Abstract

Coagulation dysfunction in critically ill patients with coronavirus disease 2019 (COVID-19) has not been well described, and the efficacy of anticoagulant therapy is unclear. In this study, we retrospectively reviewed 75 fatal COVID-19 cases who were admitted to the intensive care unit at Jinyintan Hospital (Wuhan, China). The median age of the cases was 67 (62–74) years, and 47 (62.7%) were male. Fifty patients (66.7%) were diagnosed with disseminated intra-vascular coagulation. Approximately 90% of patients had elevated D-dimer and fibrinogen degradation products, which decreased continuously after anticoagulant treatment and was accompanied by elevated albumin (all *P*<0.05). The median survival time of patients treated with anticoagulant was 9.0 (6.0–14.0) days compared with 7.0 (3.0–10.0) days in patients without anticoagulant therapy (*P*=0.008). After anticoagulation treatment, C-reactive protein levels decreased (*P*=0.004), as did high-sensitivity troponin (*P*=0.018), lactate dehydrogenase (*P*<0.001), and hydroxybutyrate dehydrogenase (*P*<0.001). In conclusion, coagulation disorders were widespread among fatal COVID-19 cases. Anticoagulant treatment partially improved hypercoagulability, prolonged median survival time, and may have postponed inflammatory processes and cardiac injury.

## INTRODUCTION

In December 2019, a pneumonia outbreak of unknown origin was found in Wuhan and quickly spread to more than 100 countries [1]. Pathogen analysis confirmed a novel enveloped RNA beta-coronavirus [2], which was named severe acute respiratory syndrome coronavirus 2 (SARS-CoV-2). The World Health Organization defined coronavirus disease 2019 (COVID-19) as a public health emergency of international concern. As of October 4, 2020, there have been 34,804,348 confirmed cases, including 1,030,738 deaths [3]. Recent research has shown that COVID-19 can not only cause pneumonia but also damage other organs such as the heart, liver, kidneys, and coagulation and immune system [4–6]. Patients suffered from critical illness often die from respiratory failure, acute respiratory distress syndrome (ARDS), shock, disseminated intra-vascular coagulation (DIC), acute renal failure, heart failure, and multiple organ dysfunction syndrome (MODS) [5, 7]. Therefore, it is particularly important to protect the lungs and other organs with treatment. According to clinical observations, we have found that the coagulation dysfunction of critical COVID-19 patients is easily induced by SARS-CoV-2. A pathological report of three COVID-19 cases by minimally invasive autopsies revealed the formation of hyaline thrombus in small vessels in both lungs and extrapulmonary organs [8]. Additionally, a recent study of risk factors associated with ARDS and death in COVID-19 patients confirmed that elevated coagulation function-related indicators (PT and D-dimer) were significantly associated with higher risk of developing ARDS [9].

Several previous studies have described the SARS-CoV-2 genome and epidemiological characteristics of COVID-19 patients [4, 10]. Patients with critical illness were characterized by rapidly progressive pneumonia, respiratory failure, and poor outcomes. COVID-19 associated coagulation dysfunction is gaining attention. A recent review proposed using low molecular weight heparin (LMWH) anticoagulant therapy for patients with severe and critical illness, although there is no clear data to confirm its efficacy [11]. This study describes the clinical and laboratory characteristics of 75 COVID-19 patients admitted to the ICU at Jinyintan Hospital of Wuhan in Hubei Province and eventually died. We also analyzed the dynamic changes of coagulation function, inflammation, and cardiac injury, and evaluated the efficacy of anticoagulation therapy in these 75 patients.

## RESULTS

### Demographic and clinical characteristics of patients

All 75 patients were confirmed to have SARS-CoV-2 infections (COVID-19, critical type) and eventually died at the Jinyintan Hospital, one of the designated hospitals for COVID-19 patients. All patients were admitted to intensive care units (ICUs) between January 20, 2020 and February 26, 2020 and died before March 10, 2020.

The median age of the patients was 67 years (IQR: 62–74), and among the 75 patients, 47 (62.7%) were male. Fifty-three patients (70.7%) had at least one preexisting chronic condition. Sixty-two patients (82.7%) received high flow oxygen therapy, 73 (97.3%) received mechanical ventilation treatment (48 [64%] received noninvasive ventilation and 61 [81.3%] received invasive ventilation), and four (5.3%) received extracorporeal membrane oxygenation (ECMO) therapy (Table 1).

**Table 1:** Characteristics of 75 fatal cases.

**Characteristic**	**Value**
**Death toll**	75
**Days from ICU to death**	8 (5-11)
**Age (yr)**	67 (62-74)
**Gender, Male**	47 (62.7%)
**Co-existing diseases**	
Hypertension	40 (53.3%)
Diabetes	17 (22.7%)
Coronary heart disease	10 (13.3%)
Chronic kidney disease	3 (4.0%)
Chronic liver disease	1 (1.3%)
Cerebrovascular disease	5 (6.7%)
Cancer	6 (8.0%)
Dysimmunity	2 (2.7%)
Respiratory diseases	5 (6.7%)
**Laboratory tests**	
WBC (×10^9^/L)	12.9 (9.4-19.2)
<4	0 (0%)
4-10	20 (26.7%)
>10	55 (73.3%)
Lymphocyte (×10^9^/L)	0.5 (0.3-0.7)
<0.8	61 (81.3%)
Neutrophil (×10^9^/L)	12.1 (8.9-18.2)
Monocyte (×10^9^/L)	0.4 (0.2-0.5)
RBC (×10^12^/L)	3.8 (3.6-4.3)
HGB (g/L)	116.0 (108.0-129.0)
PLT (×10^9^/L)	166.0 (111.0-225.0)
CRP (mg/L)	
<5	1/71 (1.4%)
5-160	33/71 (46.5%)
>160	37/71 (52.1%)
PCT (ng/mL)	
<0.05	7/73 (9.6%)
0.05-0.5	30/73 (41.1%)
>0.5	36/73 (49.3%)
D-dimer (μg/mL)	
≤1.5	8 /72 (11.0%)
1.5-10	16/72 (21.9%)
>10	49/72 (67.1%)
FDP (μg/mL)	64.7 (18.8-111.3)
>5	59/65 (90.8%)
Fbg (g/L)	4.6 (2.3-5.9)
<2	15/72 (20.8%)
2-4	14/72 (19.4%)
>4	43/72 (59.7%)
TT (s)	17.3 (16.0-19.8)
>21	12/72 (16.7%)
PT (s)	13.3 (12.4-15.3)
<13	29/72 (40.3%)
13-16	32/72 (44.4%)
>16	11/72 (15.3%)
PTA (%)	72.7 (55.8-83.9)
APTT (s)	28.1 (23.9-32.8)
AT-III (%)	90.2 (69.3-113.9)
IL-6 (pg/mL)	11.9 (8.8-20.2)
Serum ferritin (ng/mL)	
<300	1/65 (1.5%)
300-2000	36/65 (55.4%)
>2000	28/65 (43.1%)
hsTNI (pg/mL)	77.7 (27.7-223.0)
>28	53/71 (74.6%)
ALB (g/L)	27.7 (25.1-30.1)
ALT (U/L)	43.0 (24.0-68.5)
AST (U/L)	49.0 (33.5-66.5)
Cr (μmol/L)	76.4 (63.7-114.5)
>133	11/73 (15.1%)
PaO_2_/FiO_2_ (mmHg)	72.0 (60.0-113.3)
DIC (JAAM criteria)	50 (66.7%)
SOFA score	5.0 (4-7)
**Treatment**	
HFNC	62 (82.7%)
NIV	48 (64.0%)
IMV	61 (81.3%)
NIV/IMV	73 (97.3%)
ECMO	4 (5.3)

WBC: white blood cell, RBC: red blood cell, HGB: hemoglobin, PLT: platelet, FDP: fibrinogen degradation products, Fbg: fibrinogen, TT: thromboplastin time, PT: prothrombin time, PTA: PT activity, APTT: activated partial TT, AT-III: antithrombin III, hsTNI: high-sensitivity troponin, IL: interleukin, CRP: c-reactive protein, PCT: procalcitonin, ALB: albumin, ALT: alanine aminotransferase, AST: aspartate aminotransferase, Cr: creatinine, DIC: disseminated intra-vascular coagulation, JAAM: Japanese Association for Acute Medicine, SOFA: sequential organ failure assessment; HFNC: High flow nasal cannula, NIV: noninvasive ventilation, IMV: invasive mechanical ventilation, ECMO: extracorporeal membrane oxygenation.

### Laboratorial characteristics of patients

**Figure 1:**
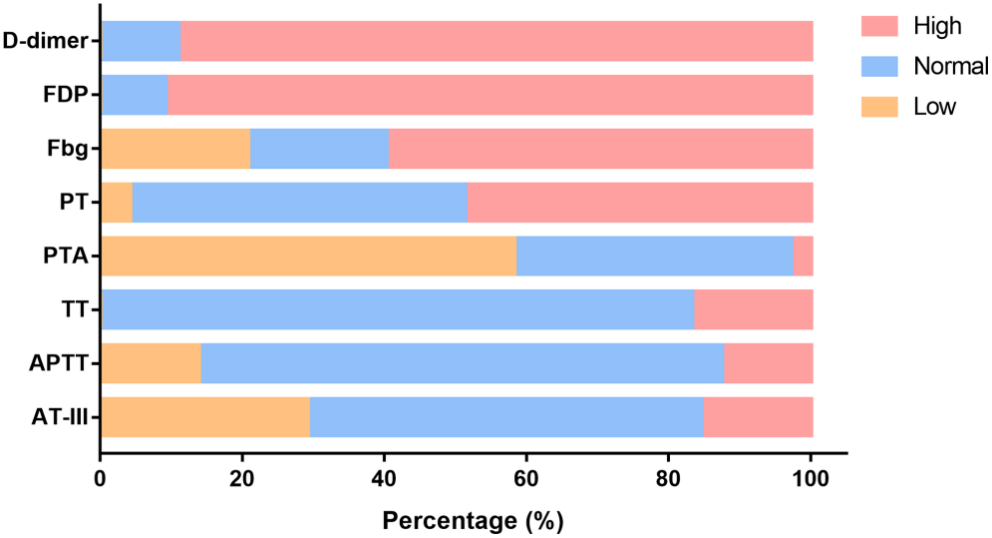
**Changes in functional coagulation laboratory markers in ICU patients with critical type COVID-19.** Percentage of patients with abnormalities in various coagulation markers. ICU: intensive care unit; COVID-19: coronavirus disease 2019. FDP: fibrinogen degradation products; Fbg: fibrinogen; PT: prothrombin time; PTA: PT activity; TT: thromboplastin time; APTT: activated partial TT; AT-III: antithrombin III.

**Figure 2:**
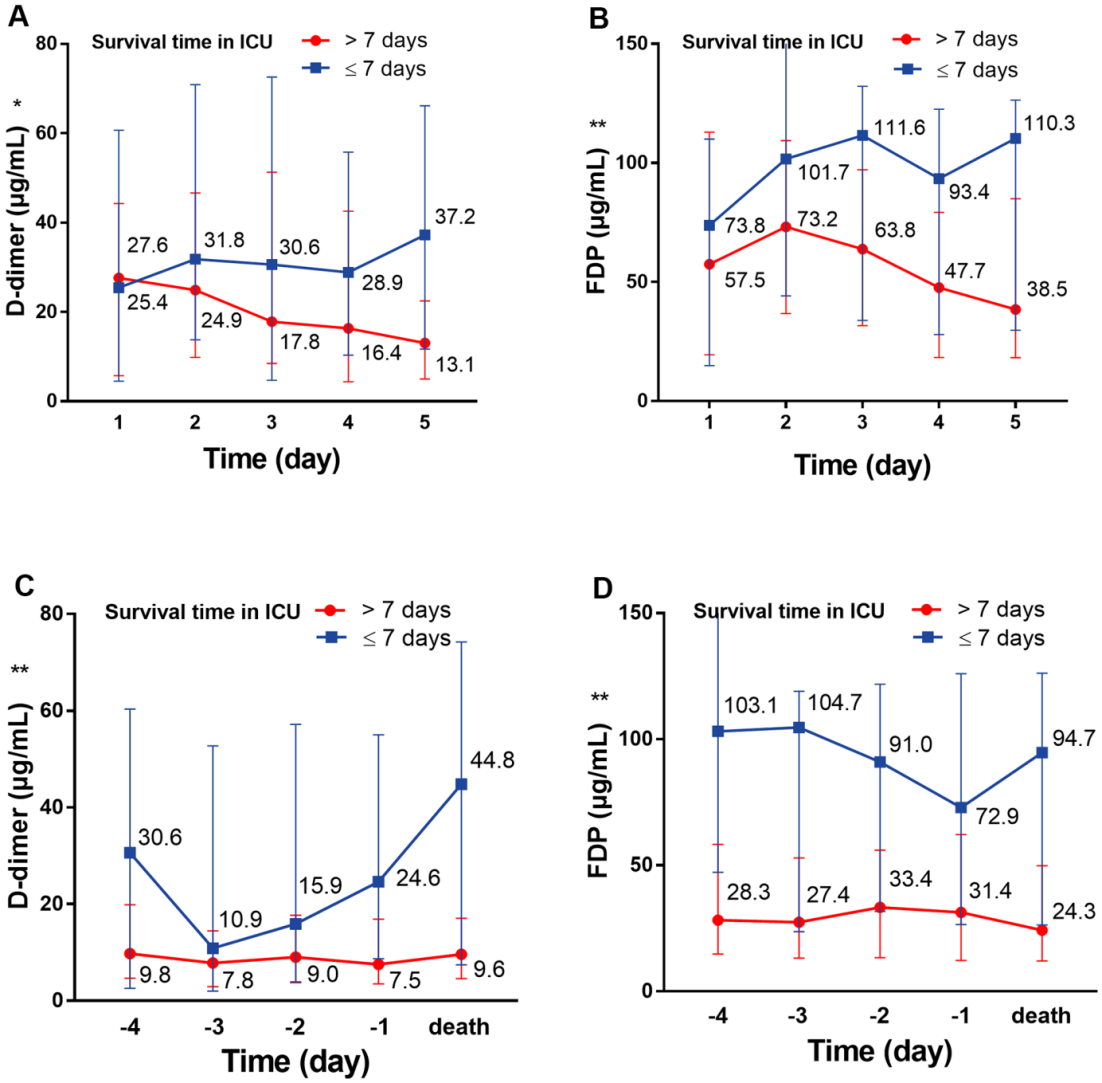
**The dynamic change of D-dimer and FDP over 5 consecutive days.** (**A**) The dynamic change of D-dimer over 5 consecutive days following ICU admission; (**B**) the dynamic change of D-dimer over 5 consecutive days before death. (**C**) The dynamic change of FDP over 5 consecutive days following ICU admission; (**D**) the dynamic change of FDP over 5 consecutive days before death. ^*^P<0.05, ^**^P<0.01 with Survival time>7 days group vs. Survival time≤7 days group, all P values can be found in Supplementary Table 1. COVID-19: coronavirus disease 2019, FDP: fibrinogen degradation products.

On the first day in the ICU, all 75 patients demonstrated increased leukocytes, with median leukocyte counts of 12.9×10^9^/L (IQR: 9.4–19.2). Lymphopenia occurred in 61 patients (81.3%) accompanied by increased neutrophils, with median neutrophil counts of 12.1×10^9^/L (IQR: 8.9–18.2). Inflammation markers were significantly increased in the majority of patients. Approximately half (36/73 [49.3%]) of the patients had serum PCT concentrations exceeding 0.5 ng/mL, and 28 of 65 (43.1%) patients had serum ferritin concentrations exceeding 2000 ng/mL. More than half (37/71 [52.1%]) of the cohort had CRP concentrations exceeding 160 mg/L; additionally, the median IL-6 concentration was 11.9 pg/mL (IQR: 8.8–20.2). Renal insufficiency was detected in 11 patients (15.1%) with serum creatinine (Cr) levels exceeding 133 μmol/L. Myocardial injury markers were abnormal in 53 patients (74.6%), including increased hsTNI levels (Table 1). Most patients had abnormal coagulation tests (Figure 1), including higher concentrations of D-dimer (89%) and FDP (90.8%), prolonged PTs (59.7%) and decreased PTA (58.3%). Two-thirds of patients (50/75) met the DIC diagnostic criteria of JAAM (Table 1). Compared with patients who survived >7 days in the ICU, the D-dimer (*P*<0.05) and FDP (*P*<0.01) levels of patients who survived <7 days gradually increased after ICU admission (Figure 2A, 2B), and remained at high levels 5 days before death (all *P*<0.01) (Figure 2C, 2D, all *P* values can be found in Supplementary Table 1).

### Baseline characteristics of patients with and without anticoagulation therapy

Thirty-five patients (46.7%) who received heparin within 3 days of ICU admission were included in the anticoagulant group; the remaining 40 patients (53.3%) were included in the non-anticoagulant group (Table 2). According to the baseline characteristics of patients in the two groups, other than the sex ratio, survival time in ICU, and IL-6 level, there were no significant differences in age, co-existing diseases, hemocytology index, functional coagulation markers, inflammatory markers, and sequential organ failure assessment (SOFA) scores between the two groups before anticoagulation treatment. Importantly, the median survival time in the anticoagulant group was longer than in the non-anticoagulant group (9.0 [IQR: 6.0–14.0] days vs. 7.0 [IQR: 3.0–10.0] days) (Table 2).

**Table 2:** Clinical characteristics between anticoagulant and non-anticoagulant patients.

	**Non-anticoagulant (n=40)**	**Anticoagulant (n=35)**	***P***
Age (yr)	67 (61-75)	66 (62-72)	0.610
Gender, Male	30 (75%)	17 (48.6%)	**0.018**
Days from ICU to death	7 (3-10)	9 (6-14)	**0.008**
Co-existing diseases			
0	12 (30%)	10 (28.6%)	0.959
1	14 (35%)	13 (37.1%)	
2	9 (22.5%)	8 (22.9%)	
3	5 (12.5%)	3 (8.6%)	
4	0 (0%)	1 (2.9%)	
**Laboratory tests**			
WBC (×109/L)	12.4 (9.3-17.8)	14.2 (11.0-19.7)	0.588
Lymphocyte (×109/L)	0.5 (0.3-0.6)	0.5 (0.3-0.7)	0.686
Neutrophil (×109/L)	11.7 (8.6-16.4)	13.1 (9.9-18.4)	0.663
Monocyte (×109/L)	0.3 (0.2-0.5)	0.4 (0.2-0.5)	0.534
RBC (×1012/L)	3.9 (3.6-4.3)	3.8 (3.3-4.1)	0.137
HGB (g/L)	118 (109-134)	114 (101-126)	0.074
PLT (×109/L)	170 (105-227)	165 (118-225)	0.932
CRP (mg/L)			
>160	21/38 (55.3%)	16/33 (48.5%)	0.569
PCT (ng/mL)			
>0.5	20/39 (51.3%)	16/34 (47.1%)	0.719
D-dimer (μg/mL)			
>10	26/40 (65.0%)	23/33 (70.0%)	0.671
FDP (μg/mL)	73.8 (25.3-113.7)	63.3 (16.8-107.7)	0.580
Fbg (g/L)	4.6 (2.1-6.1)	4.6 (2.4-5.9)	0.804
TT (s)	17.2 (16.0-20.4)	17.5 (15.8-19.5)	0.865
PT (s)	13.9 (12.3-15.9)	13.0 (12.4-14.6)	0.218
PTA (%)	65.9 (51.1-83.6)	73.6 (58.7-85.5)	0.188
APTT (s)	28.4 (24.3-33.6)	27.3 (22.2-31.0)	0.249
AT-III (%)	90.2 (69.7-122.5)	89.5 (67.5-109.3)	0.563
IL-6, (pg/mL)	10.4 (8.1-13.7)	14.3 (9.8-23.2)	**0.039**
Serum ferritin (ng/mL)			
>2000	14/37 (37.8%)	14/28 (50.0%)	0.327
hsTNI (pg/mL)	65.3 (14.0-530.4)	103.2 (35.1-201.2)	0.729
LDH (U/L)	612.5 (457.0-919.5)	642.0 (424.3-779.8)	0.586
HBDH (U/L)	512.5 (357.3-720.3)	519.5 (306.3-665.0)	0.486
CK (U/L)	132.5 (57.3-264.3)	112.5 (64.3-219.8)	0.592
CK-MB (U/L)	18.0 (12.8-28.5)	20.5 (14.5-25.8)	0.846
ALB (g/L)	27.5 (25.5-29.9)	28.0 (24.9-30.7)	0.736
ALT (U/L)	45.5 (25.8-70.3)	35.0 (19.0-65.0)	0.224
AST (U/L)	55.0 (34.8-67.5)	48.0 (32.0-61.0)	0.342
Cr (μmol/L)	81.2 (65.5-118.3)	72.5 (63.4-101.0)	0.337
DIC (JAAM criteria)	29 (72.5%)	21 (60%)	0.252
PaO2/FiO2 (mmHg)	69.0 (59.0-113.0)	78.0 (65.0-115.0)	0.318
SOFA score	5.0 (4.0-7.0)	5.0 (4.0-6.0)	0.420
**Treatment**			
HFNC	35 (87.5%)	27 (77.1%)	0.237
NIV	27 (67.5%)	21 (60.0%)	0.500
IMV	28 (70.0%)	33 (94.3%)	**0.007**
ECMO	2 (5.0%)	2 (5.7%)	1.000

WBC: white blood cell, RBC: red blood cell, HGB: hemoglobin, PLT: platelet, CRP: c-reactive protein, PCT: procalcitonin, FDP: fibrinogen degradation products, Fbg: fibrinogen, TT: thromboplastin time, PT: prothrombin time, PTA: PT activity, APTT: activated partial TT, AT-III: antithrombin III, IL: interleukin, hsTNI: high-sensitivity troponin, LDH: lactic dehydrogenase, HBDH: hydroxybutyrate dehydrogenase, CK: creatine kinase, CK-MB: creatine kinase-MB, ALB: albumin, ALT: alanine aminotransferase, AST: aspartate aminotransferase, Cr: creatinine, DIC: disseminated intra-vascular coagulation, JAAM: Japanese Association for Acute Medicine, SOFA: sequential organ failure assessment; HFNC: High flow nasal cannula, NIV: noninvasive ventilation, IMV: invasive mechanical ventilation, ECMO: extracorporeal membrane oxygenation.

### Dynamic changes in coagulation, inflammation, and cardiac injury markers within 5 days after anticoagulation treatment

**Figure 3:**
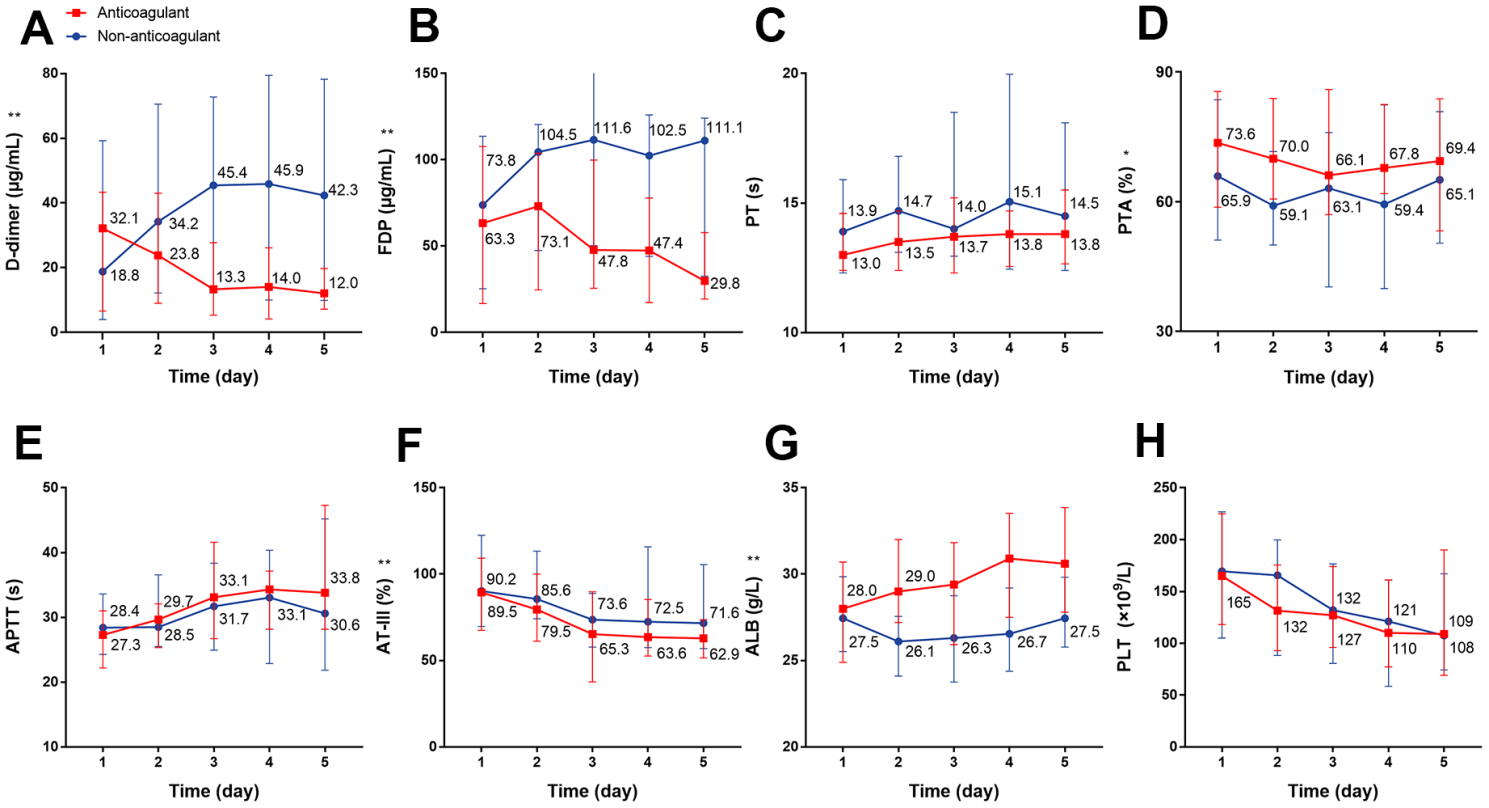
**The dynamic changes in coagulation function over 5 consecutive days using laboratory markers in critical type COVID-19 patients with or without anticoagulation treatment.** (**A**) D-dimer (**B**) FDP (**C**) PT (**D**) PTA (**E**) APTT (**F**) AT-III (**G**) ALB (**H**) PLT. ^*^P<0.05, ^**^P<0.01 with anticoagulant group vs. non-anticoagulant group, all P values can be found in Supplementary Table 2. COVID-19: coronavirus disease 2019, FDP: fibrinogen degradation products, PT: prothrombin time, PTA: PT activity, APTT: activated partial thromboplastin time, AT-III: antithrombin III, ALB: albumin, PLT: platelet.

Next, we analyzed the dynamic changes in coagulation markers through a 5-day period in response to anticoagulant treatment compared with the non-anticoagulant group. The results showed that the D-dimer concentration was significantly and continuously deceased after heparin use; conversely, the D-dimer concentration was continuously increased during the 5 days in patients of the non-anticoagulant group (*P*=0.007) (Figure 3A). We also observed significantly decreased FDP (*P*<0.001) and AT-III (*P*=0.001), and increased PTA (*P*=0.022) and ALB (*P*<0.001) in the anticoagulant group (Figure 3B, 3D, 3F, 3G). There were no obvious dynamic differences in PT, APTT, or PLT (all *P*>0.05) (Figure 3C, 3E, 3H, all P values can be found in Supplementary Table 2).

**Figure 4:**
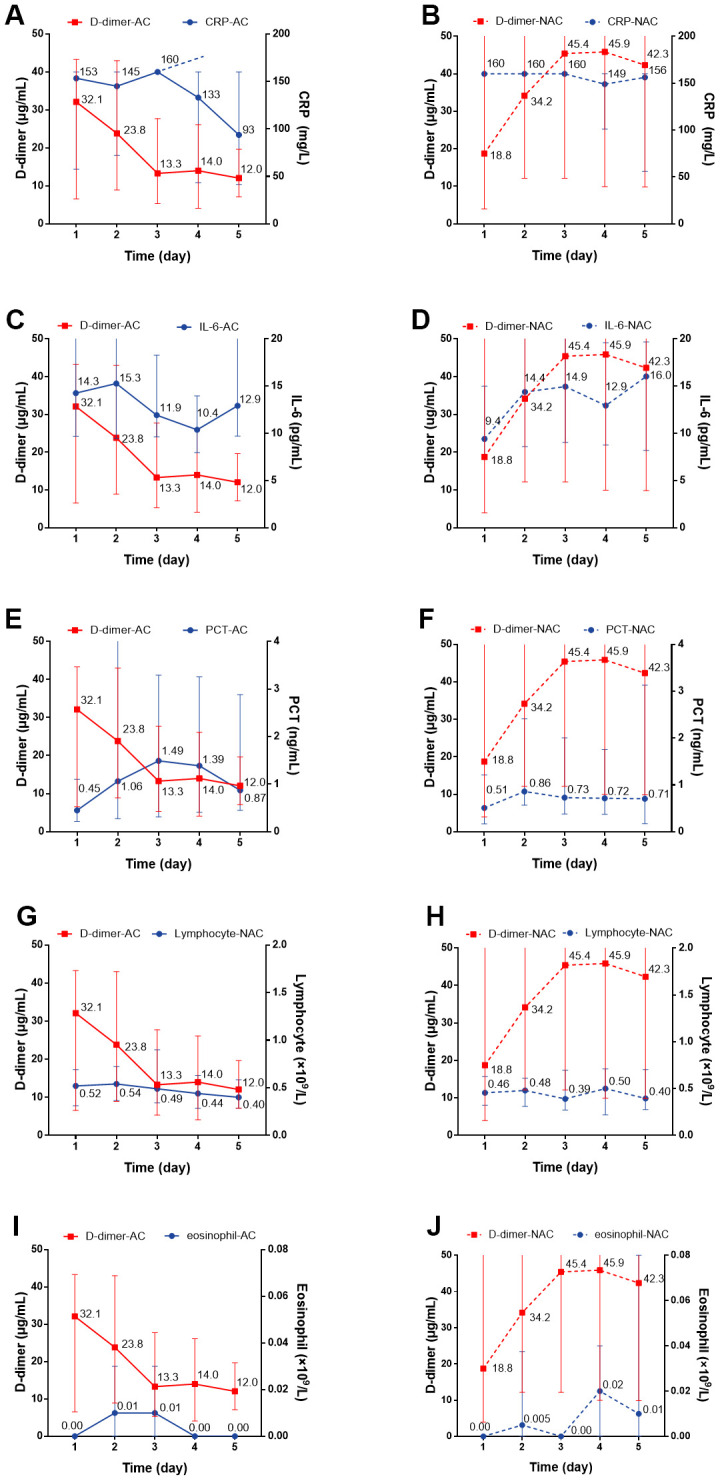
**The dynamic changes over 5 consecutive days in inflammatory markers in critical type COVID-19 patients with or without anticoagulation treatment.** (**A**, **B**) CRP; (**C**, **D**) IL-6; (**E**, **F**) PCT (**G**, **H**) Lymphocyte; (**I**, **J**) Eosinophil. All *P* values can be found in Supplementary Table 2. COVID-19: coronavirus disease 2019, NAC: non-anticoagulant; AC: anticoagulant, CRP: c-reactive protein, IL-6: Interleukin-6, PCT: procalcitonin.

**Figure 5:**
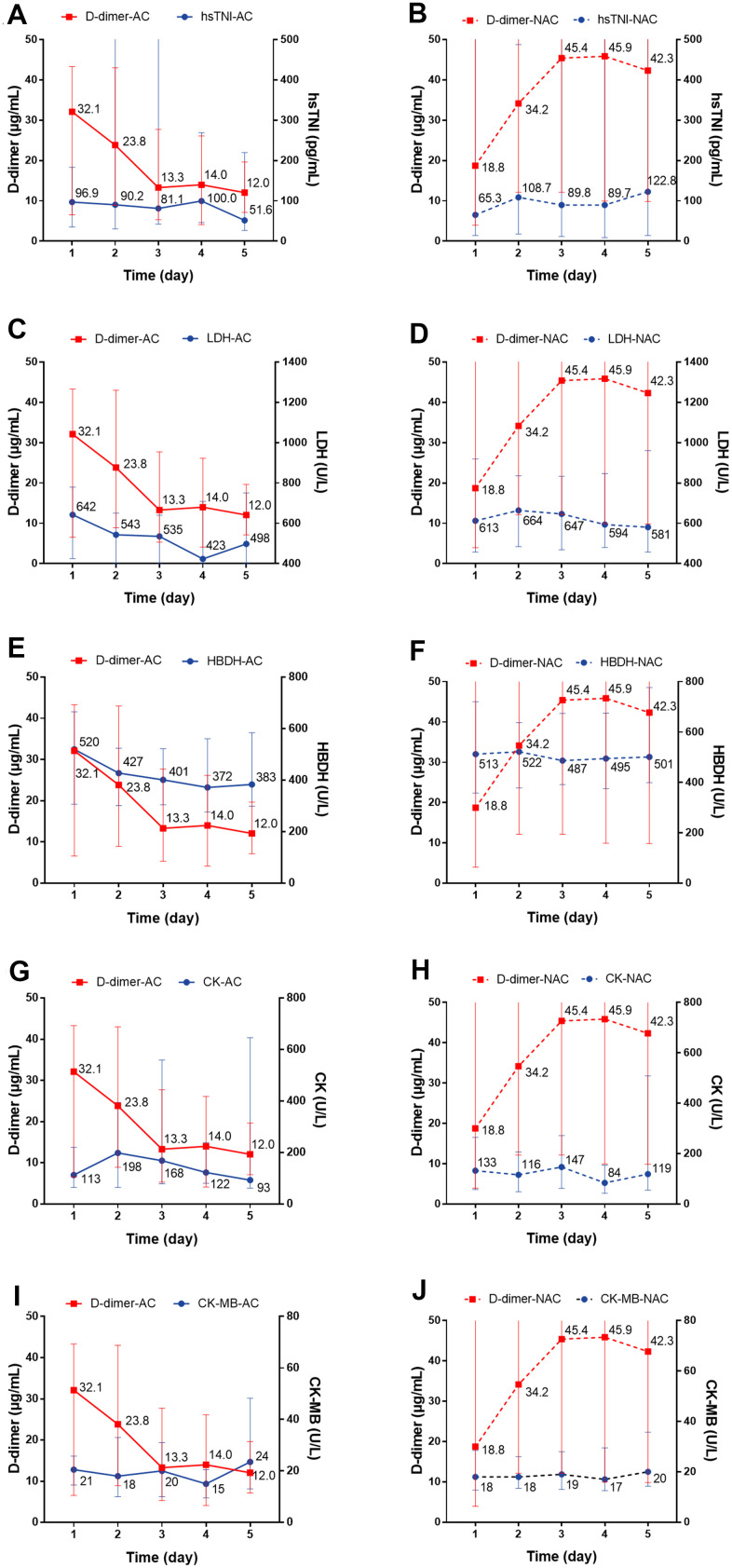
**The dynamic changes over 5 consecutive days in myocardial markers in critical type COVID-19 patients with or without anticoagulation treatment.** (**A**, **B**) hsTNI; (**C**, **D**) LDH; (**E**, **F**) HBDH; (**G**, **H**) CK; (**I**, **J**) CK-MB. All *P* values can be found in Supplementary Table 2. COVID-19: coronavirus disease 2019, NAC: non-anticoagulant; hsTNI: high-sensitivity troponin, LDH: lactic dehydrogenase, HBDH: hydroxybutyrate dehydrogenase, CK: creatine kinase, CK-MB: creatine kinase-MB.

We individually evaluated the dynamic changes of inflammatory markers with D-dimer within 5 days. For the anticoagulant group, we observed a dynamic decrease over a 5-day period in the concentration of CRP with decreased D-dimer level, while for non-anticoagulant group, the concentration remained at a high level (*P*=0.004) (Figure 4A, 4B). There were no significant dynamic changes in serum IL-6, PCT, lymphocyte, or eosinophil levels in patients with or without anticoagulation therapy (Figure 4C–4J). Regarding cardiac injury indictors, we found a significant decrease in hsTNI (*P*=0.018), LDH (*P*<0.001), and HBDH (*P*<0.001) in patients with anticoagulation therapy (Figure 5A–5F; all *P* values can be found in Supplementary Table 2).

## DISCUSSION

In this observational study, we report the clinical and laboratory characteristics of 75 patients who died in ICUs from COVID-19. All patients were seriously ill (critical type) at admission, and the condition of some patients deteriorated rapidly, suggesting SARS-CoV-2 infection may lead to poor outcomes in critically ill patients. It was remarkable that different degrees of coagulation dysfunction could be observed in COVID-19 patients, and it was particularly significant in critical type ICU patients. SARS-CoV-2 infects human cells *via* angiotensin-converting enzyme 2 (ACE2) [12]. ACE2 is expressed in alveolar epithelial cells, vascular endothelial cells, and the immune system at different levels [13]. SARS-CoV-2 can be rapidly recognized after entering the body, which activates the innate immune system to clear the virus; however, excessive activation can cause a cytokine storm, damage the microvasculature by direct and indirect means [14], activate the coagulation system, and inhibit fibrinolysis and the anticoagulation system. The resulting extensive thrombosis in microvessels often leads to poor outcomes [15].

Studies have confirmed the high risk of thrombosis in COVID-19 patients [16, 17], and it has been reported that approximately half of COVID-19 patients have elevated D-dimer levels during disease progression; D-dimer levels have also been found to be significantly higher in patients with severe illness [18]. Increased D-dimer levels have become an independent risk factor for death in COVID-19 patients [19]. A similar phenomenon was demonstrated in our study, in which almost all the deceased patients showed coagulation dysfunction during the course of the disease, especially the high levels of D-dimer. Two-thirds of the patients met diagnostic criteria for DIC (JAAM), and approximately 90% of the patients had D-dimer levels >1.5 μg/mL when they were admitted to the ICU. A concurrent study confirmed that 25% (20/81) of severe patients underwent venous thromboembolism (VTE) during hospitalization, demonstrating that 1.5 μg/mL is an appropriate cut-off value to reflect the high prevalence of thrombosis in COVID-19 patients [20]. Additionally, COVID-19-related coagulation dysfunction is a dynamically changing process. We noticed that the dynamic changes in coagulation function after ICU admission, especially the continuously elevated D-dimer and FDP levels, may be associated with reduced survival time in ICU. Early identifying them and continuously monitoring the trend will predict clinical prognoses.

In total, 35/75 (46.7%) patients in this study received anticoagulant therapy (LMWH or enoxaparin) within 3 days of ICU and admission, followed by 5 days of dynamic monitoring. Compared with patients in the non-anticoagulant group, the median survival time in ICU was significantly longer for the anticoagulant group, although all patients in both groups eventually died. Additionally, we found that the dynamic changes in coagulation markers such as D-dimer, FDP, and PTA in the anticoagulant group were partially improved compared with the non-anticoagulation group. ALB has been reported to have significant anticoagulant action *in vitro* [21] and was negatively related to the risk of thrombosis [22, 23]. In this study, we found that ALB gradually increased after anticoagulation treatment. These data showed that anticoagulant treatment effectively relieved hypercoagulability in COVID-19 patients.

Classically, the association between coagulation and inflammation has been regarded as a crosstalk process [24, 25]. During inflammatory reactions, inflammation mediators are released, which activate blood coagulation and consume mass clotting factors through the ‘waterfall sample cascade,’ which may lead to blood coagulation disorders [26]. Meanwhile, some key components of the coagulation system can promote inflammation through direct and indirect mechanisms, such as tissue factor and fibrinogen, which are not only key in the coagulation process, but also have multiple roles in tissue damage and inflammation [25, 27]. A recent study reported the vital role of inflammation in COVID-19 progression [28, 29]. Here, we analyzed the dynamic changes of some inflammatory markers after anticoagulant treatment and found improved CRP expression after anticoagulant therapy, which is a frequent prognostic factor for COVID-19 that reflects the inflammatory process [30]. Moreover, the dynamic improvement of cardiac injury indictors such as hsTNI, LDH, and HBDH were further demonstrated. As the most common complication of COVID-19, cardiac injury shows viral load in the myocardium and is closely related to regional and systemic inflammatory states [31, 32]. Anticoagulant therapy could relieve hypercoagulability and prevent and improve the formation of systemic microthrombi, including coronary microvascular thrombosis [33, 34]. However, this recovery did not reverse the outcome of patients in this study, who had severe multiple organ failure including respiratory and other organ dysfunctions, although our results showed partial improvement in inflammation and heart damage. Dynamically monitoring levels of D-dimer and indicators of inflammation and cardiac injury could assess the efficacy of anticoagulant therapy and severity of systemic disease status. After an accurate thrombosis risk assessment, more aggressive anticoagulation strategies may be needed in early rather than in late disease stages to improve outcomes.

This study had some limitations and raises areas for further study. One of the limitations of the study was the small sample size. Interpretations of our findings might be limited due to its retrospective nature with the possible loss of data. To overcome this limitation, a prospective study design and complete data collection would be needed. Additionally, at the beginning of the epidemic, due to the serious shortage of medical resources and staff, dynamic monitoring of patient conditions was insufficient, and some patients with coagulation disorders could not receive comprehensive screening, such as vascular ultrasound and CT, according to our data at the time.

## CONCLUSIONS

Coagulation disorders were widespread in critical COVID-19 patients in ICUs. According to our data, two-thirds of fatal patients were diagnosed with DIC upon ICU admission. In critically ill patients, anticoagulant treatment partially improved hypercoagulability, prolonged median ICU survival time, and potentially postponed inflammation and cardiac injury.

## MATERIALS AND METHODS

### Study design and data collection

This retrospective study included 75 patients (≥18-years-old) who were admitted to the ICU at Jinyintan Hospital (Wuhan, China) with SARS-CoV-2 infections between January 20 and February 26, 2020 and died before March 10, 2020. All patients were diagnosed with COVID-19 (critical type) according to The WHO interim guidance and Chinese management guidelines for COVID-19 (version 6.0) [35, 36]. Patients’ epidemiology, demographics, clinical characteristics, laboratory and treatment data were obtained from the standard electronic medical record system. All data were collated by two researchers, and then checked and confirmed by two physicians. This study was approved by the Research Ethics Commission of Jinyintan Hospital (KY-2020–56.01).

### Laboratory procedures

The data for complete blood count, coagulation function tests (including D-dimer, fibrinogen degradation products [FDP], prothrombin time [PT], PT activity [PTA], activated partial thromboplastin time [APTT], fibrinogen [Fbg], Antithrombin III [AT-III], and platelet [PLT]), serum inflammation markers (including interleukin-6 [IL-6], serum ferritin, C-reactive protein [CRP], and procalcitonin [PCT]), and other serum biochemical tests (including renal and liver function markers, albumin [ALB], high-sensitivity troponin [hsTNI], lactic dehydrogenase [LDH], and hydroxybutyrate dehydrogenase [HBDH]) were collected for each patient. All clinical laboratory data were generated by the clinical laboratory of Jinyintan hospital. Ultrasonography and radiological examinations were also performed for patients.

### Definition

The severity status of COVID-19 was defined according to the Chinese management guidelines for COVID-19 (version 7.0) [36]. DIC was defined according to the scoring algorithm criteria established by the Japanese Association for Acute Medicine (JAAM) [37]. Anticoagulant therapy was defined as the use of LMWH (100 U/kg weight per 12 h) or enoxaparin (40 mg per day) within 3 days of the patient's admission to the ICU and the duration was not less than 5 days or until death.

### Statistical analysis

Continuous data are presented as median (IQR) and were compared by the Mann-Whitney U test. Categorical data are presented as counts (percentages) and were compared by the Chi-square test or Fisher’s exact test. To compare the dynamic changes in clinical indictors of patients over 5 consecutive days, a generalized linear mixed model was used. SPSS 23.0 and GraphPad Prism 7.0 were used for analyses. P<0.05 was considered statistically significant.

## Supplementary Material

Supplementary Tables
