# Supplementary material for: Coagulation dysfunction in ICU patients with coronavirus disease 2019 in Wuhan, China: a retrospective observational study of 75 fatal cases
Source: Aging (Albany NY). 2020 Dec 9;13(2):1591–607. doi: 10.18632/aging.202223 (PMC7880373; doi:10.18632/aging.202223)
Supplement: Supplementary Tables [file aging-13-202223-s001.pdf]

SUPPLEMENTARY TABLES

Supplementary Table 1. Statistic process of data from Figure 2.

Indicator	Model term	Coefficient	SE	t	*P*	95% CI	
						Lower	Upper
The dynamic change of indictors for 5 consecutive days since ICU admission							
D-dimer	Threshold[a] (D-dimer≤10)	-0.498	0.257	-1.935	0.054	-1.004	0.009
	Threshold[a] (80≥D-dimer>10)	2.282	0.302	7.550	0.000	1.687	2.878
	Group A[b]	0.514	0.245	2.096	**0.037**	0.031	0.996
	Group B	0	-	-	-	-	-
PFDP	Intercept	72.073	21.796	3.307	0.001	29.145	115.001
	Group A[b]	72.093	19.721	3.656	**0.000**	33.253	110.934
	Group B	0	-	-	-	-	-
The dynamic change of indictors for 5 consecutive days before death							
D-dimer	Threshold[a] (D-dimer≤10)	0.131	0.299	0.438	0.662	-0.457	0.719
	Threshold[a] (80≥D-dimer>10)	2.638	0.348	7.570	0.000	1.952	3.324
	Group A[b]	1.243	0.248	5.021	**0.000**	0.756	1.731
	Group B	0	-	-	-	-	-
PFDP	Intercept	70.710	26.814	2.637	0.009	17.914	123.507
	Group A[b]	105.539	19.976	5.283	**0.000**	66.206	144.872
	Group B	0	-	-	-	-	-

Group A means patients whose survival time in ICU ≤ 7 days; Group B means patients whose survival time in ICU>7 days.
[a]Compared with D-dimer >80 ug/mL; [b]Compared with Group B.

Supplementary Table 2. Statistic process of data from Figure 3, Figure 4 and Figure 5.

Indicator	Model term	Coefficient	SE	t	P	95% CI	
						Lower	Upper
D-dimer	Threshold[a] (D-dimer≤10)	-0.385	0.265	-1.450	0.148	-0.907	0.138
	Threshold[a] (80>D-dimer> 10)	2.419	0.314	7.696	0.000	1.800	3.037
	NAC group[b]	0.667	0.244	2.729	**0.007**	0.186	1.149
	AC group	0	-	-	-	-	-
PFDP	Intercept	63.053	21.803	2.892	0.004	20.111	105.994
	NAC group[b]	82.003	19.375	4.232	**0.000**	43.844	120.163
	AC group	0	-	-	-	-	-
PTA	Intercept	76.171	3.612	21.087	0.000	69.061	83.281
	NAC group[b]	-6.735	2.915	-2.310	**0.022**	-12.474	-0.997
	AC group	0	-	-	-	-	-
PT	Intercept	12.894	2.230	5.782	0.000	8.505	17.284
	NAC group[b]	3.731	1.955	1.908	0.057	-0.117	7.579
	AC group	0	-	-	-	-	-
APTT	Intercept	30.210	3.777	7.998	0.000	22.776	37.645
	NAC group[b]	1.047	3.312	0.316	0.752	-5.471	7.566
	AC group	0	-	-	-	-	-
AT3	Intercept	88.992	4.418	20.141	0.000	80.290	97.694
	NAC group[b]	11.717	3.565	3.287	0.001	4.696	18.739
	AC group	0	-	-	-	-	-
ALB	Intercept	28.353	0.601	47.214	0.000	27.171	29.534
	NAC group[b]	-2.387	0.455	-5.243	**0.000**	-3.282	-1.491
	AC group	0	-	-	-	-	-
PLT	Intercept	167.162	10.855	15.400	0.000	145.805	188.520
	NAC group[b]	-4.022	8.382	-0.480	0.632	-20.515	12.471
	AC group	0	-	-	-	-	-
CRP	Threshold[c] (CRP≤100)	-0.667	0.277	-2.409	0.017	-1.212	-0.122
	Threshold[c] (100<CRP≤160)	0.306	0.275	1.113	0.267	-0.235	0.847
	NAC group[b]	0.648	0.226	2.866	**0.004**	0.203	1.093
	AC group	0	-	-	-	-	-
IL-6	Intercept	26.099	5.634	4.632	0.000	14.995	37.204
	NAC group[b]	-1.187	7.479	-0.159	0.874	-15.926	13.553
	AC group	0	-	-	-	-	-
PCT	Intercept	1.992	1.266	1.574	0.117	-0.499	4.483
	NAC group[b]	1.602	0.982	1.632	0.104	-0.330	3.534
	AC group	0	-	-	-	-	-
Lymphocyte	Intercept	0.480	0.066	7.227	0.000	0.350	0.611
	NAC group[b]	0.018	0.050	0.364	0.716	-0.081	0.117
	AC group	0	-	-	-	-	-
Eosnophil	Intercept	0.016	0.009	1.791	0.074	-0.002	0.033
	NAC group[b]	-0.005	0.007	-0.794	0.428	-0.018	0.008
	AC group	0	-	-	-	-	-
hsTNI	Intercept	520.049	648.273	0.802	0.423	-756.12	1796.22
	NAC group[b]	1311.733	553.327	2.371	**0.018**	222.473	2400.99

	AC group	0	-	-	-	-	-
LDH	Intercept	720.507	68.395	10.535	0.000	585.876	855.137
	NAC group[b]	202.730	52.798	3.840	**0.000**	98.800	306.659
	AC group	0	-	-	-	-	-
HBDH	Intercept	533.436	32.835	16.246	0.000	468.801	598.071
	NAC group[b]	91.438	25.166	3.633	0.000	41.899	140.977
	AC group	0	-	-	-	-	-
CK	Intercept	149.424	78.436	1.905	0.058	-4.975	303.822
	NAC group[b]	-69.764	59.555	-1.171	0.242	-187.00	47.468
	AC group	0	-	-	-	-	-
CK-MB	Intercept	23.815	3.021	7.882	0.000	17.868	29.762
	NAC group[b]	2.920	2.628	1.111	0.267	-2.253	8.092
	AC group	0	-	-	-	-	-

NAC: Non-anticoagulant; AC: Anticoagulant; Other abbreviations was shown in Table 1.
[a]Compared with D-dimer >80 ug/mL; [b]Compared with AC group.
[c]Compared with CRP>160 mg/L.[d] Compared with ferritin>2000 ng/mL.
